# *Ophiocordyceps sinensis*: Antioxidant, Proteolytic Activities and Synthesis of Silver Nanoparticles

**DOI:** 10.3390/life16071052

**Published:** 2026-06-24

**Authors:** Anna Uhrinová, Lucia Ungvarská Maľučká, Martin Pavlík, Ľudmila Tkáčiková, Miriam Bačkorová

**Affiliations:** 1 Department of Chemistry, Biochemistry and Biophysics, University of Veterinary Medicine and Pharmacy in Košice, Komenského 73, 041 81 Košice, Slovakia; anna.uhrinova@uvlf.sk (A.U.); lucia.ungvarska.malucka@uvlf.sk (L.U.M.); 2 Department of Chemical Drugs, Faculty of Pharmacy, Masaryk University, Palackého třída 1946/1, 612 00 Brno, Czech Republic; 3 Department of Integrated Forest and Landscape Protection, Faculty of Forestry, Technical University in Zvolen, T.G. Masaryka 24, 960 01 Zvolen, Slovakia; pavlik@tuzvo.sk; 4 Department of Microbiology and Immunology, University of Veterinary Medicine and Pharmacy in Košice, Komenského 73, 041 81 Košice, Slovakia; ludmila.tkacikova@uvlf.sk; 5 Department of Pharmaceutical Technology, Pharmacognozy and Botany, University of Veterinary Medicine and Pharmacy in Košice, Komenského 73, 041 81 Košice, Slovakia

**Keywords:** antioxidant and proteolytic activity, extractions, *Ophiocordyceps sinensis*, silver nanoparticles

## Abstract

Natural *Ophiocordyceps sinensis* is a highly valued medicinal fungus known for its antitumor, immunomodulatory, and antiviral properties. Due to extensive overharvesting in Asia, cultivated alternatives have become increasingly important. This study aimed to evaluate the biological activity and chemical composition of extracts obtained from cultivated *Ophiocordyceps sinensis* grown using different rice substrates. Methanolic extracts were prepared from solid-state cultivated *Ophiocordyceps sinensis* grown on *Oryza sativa* var. *indica* and *Oryza sativa* var. *japonica*. Antioxidant activity was determined using the DPPH assay, while proteolytic activity was evaluated with the azocasein substrate. Chemical characterization of major compounds was performed using ^1^D and ^2^D NMR spectroscopy, together with IR spectroscopy. UV/Vis spectrophotometry was employed to confirm the formation of silver nanoparticles in AgNO_3_ solution. Antimicrobial activity was tested against bacterial strains, including *Escherichia coli* and *Staphylococcus aureus*. All prepared methanolic extracts exhibited measurable antioxidant and proteolytic activities. The dominant identified compounds were *Z*-oleic acid, linoleic acid, and D-mannitol. Selected extracts successfully induced the formation of silver nanoparticles. The highest antimicrobial activity against *Escherichia coli* was observed for sample 1OS, reaching a mean % RIZD value of 129.32 ± 0.58%.

## 1. Introduction

*Cordyceps* is a rare, exotic, and medicinal parasitic fungus that has been part of traditional Chinese medicine for centuries, with the species *Ophiocordyceps sinensis* (OS) currently being the most widely studied. The name *Cordyceps* encompasses a group of Ascomycetes that have evolved as endoparasites, mainly on arthropods, and as symbionts of ascomycete fungi of the genus *Elaphomyces* [1]. The name ‘Chinese cordyceps’ is used in reference to the complex of the species *Ophiocordyceps sinensis* and the ghost moth caterpillar *Thitarodes armoricanus*. In 1878, the Italian scholar Saccardo named the *Cordyceps* obtained from China *Cordyceps sinensis* (Berk.) Sacc., but its current correct name is *Ophiocordyceps sinensis* (Berk.) G. H. Sung, J. M. Sung, Hywel-Jones & Spatafora (2007) (Ophiocordycipitaceae, Hypocreales, Sordariomycetes, Ascomycota) [2,3,4].

Natural OS is extremely valuable for its anticancer, immunomodulatory, antibacterial, anti-inflammatory, and antiviral effects [5,6,7]. Due to overexploitation, host specificity, and an inability to spread to other geographical areas, its high price and overall scarcity on the market remain major issues. The ongoing environmental damage caused by its harvesting is also a serious concern. To meet the high global demand for this fungus, artificial cultivation has been initiated, primarily in the form of mycelium but also as fruiting bodies [8,9]. Biotechnological processes are now able to replicate the natural conditions under which *Cordyceps* grows, to such an extent that cultivated OS is becoming a viable substitute for the natural fungus. Current cultivation technologies enable the production of *Cordyceps* with improved quality, consistency, and scalability.

A study by Zhou et al. [10] clearly demonstrated that the mean levels of adenosine and cordycepin in Chinese *Cordyceps* were significantly higher, while the mean levels of mannitol and polysaccharides were significantly lower in the cultivated type than in the natural type. No significant difference in the mean soluble protein content was observed. Furthermore, there was no difference in the chemical constituents detected between cultivated and natural Chinese *Cordyceps* [11]. Extracts from both cultivated and natural mycelia exhibit direct and potent antioxidant effects [12]. This research suggests that the major nutritional composition of cultivated and natural *Cordyceps* is identical, and the cultivated type can be used as an effective substitute.

Another important topic in biotechnology is silver nanoparticles (Ag NPs). These are currently proving to be highly advantageous antibacterial agents with applications in the cosmetic, medical, and textile industries [13,14]. Thus, much attention is turning to the biosynthesis of silver nanoparticles in conjunction with biological material. This is most commonly achieved with various species of bacteria, fungi, yeasts, algae and, not least, various medicinally important plant species. The greatest advantage of using these biomaterials is their predominant non-toxicity, acting as reducing and stabilizing agents [15]. On the other hand, silver, due to its unique properties, enhances the beneficial effects of important biological substances contained in biomaterials.

Fungi can be considered as organisms that can adapt very well to their environment and/or adapt rapidly to changes that take place in their environment. They contain a large number of enzymes and bioactive compounds such as polysaccharides, organic acids, and many trace elements. Among these, polysaccharides act as reducing agents and allow the reduction of silver itself [16]. Currently, some fungal species are already being used for the synthesis of silver nanoparticles, particularly *Aspergillus clavatus*, *Penicillium citrinum*, *Pleurotus* sp., and *Schizophyllum commune* [17,18]. Thus, the synthesis of nanoparticles in combination with natural substances may provide a new source of pharmaceutically important preparations [19,20,21]. Due to the above-mentioned facts, there are currently limited data regarding how rice subspecies used as solid substrates influence antioxidant activity, proteolytic activity, and the ability of cultivated OS to synthesize Ag NPs with antimicrobial properties.

## 2. Materials and Methods

### 2.1. Cultivation of Ophiocordyceps sinensis

Rice is widely available, inexpensive, and possesses both a suitable structure and an appropriate carbon-to-nitrogen (C:N) ratio, while being easily digestible for humans. The use of other, cheaper substrates is problematic in this case. Cheap bran or soy have a high nitrogen content, but their structure becomes unsuitable after soaking. Wheat bran, when moistened, loses its structure and forms a compact, poorly aerated substrate. This leads to inhibited mycelial growth and anaerobic decomposition. Too much nitrogen in both bran and soybeans has a toxic effect on the mycelium and, paradoxically, stops its growth.

We tested the two most globally accessible rice standards: the *Indica* variety and the *Japonica* variety. We aimed to determine whether *Ophiocordyceps sinensis*, during solid-state fermentation, prefers a loose, firmer substrate with a slow release of sugars (*Indica* long-grain variety) or a softer, stickier substrate with readily available energy (*Japonica* short-grain variety) for its growth [22,23]. The resulting mycelium, obtained through the solid-state fermentation process and fully colonized within the rice, can be processed in its entirety into the final product (e.g., capsule powder), as rice has a neutral taste and is easily digestible for humans. If bran was used as a base, the product would contain a high proportion of indigestible wheat fibre, which would reduce the concentration of active substances per gram of the final powder.

Production strains of OS were available in our research. We used these in the inoculation of two rice varieties (Table 1). Proper sample preparation is very important and can significantly impact the results of a wide number of analyses. In the current research, production strains of the fungus OS were used, provided to us by the supplier MykoForest Company: Martin Rajtar (Velčice, Slovakia). They are part of the MykoForest Type Culture Collection and were originally obtained from the supplier Aloha Medicinals (Carson City, NV, USA).

The cultures were then cultivated in Petri dishes containing Malt extract agar (MYA) medium (20 g of malt extract, 2 g of yeast extract and 20 g of agar per litre of distilled water; product of Biolife Italiana s.r.l., Milan, Italy) at 20 °C in complete darkness for 14 days [24].

A 100 g sample of rice was soaked in distilled water. Following centrifugation, the resulting 180 g of conditioned rice was transferred into a borosilicate glass jar equipped with a PTFE filter. To this substrate, 200 mL of a specific nutrient solution was added. This nutrient solution was prepared by dissolving yeast extract (8 g L^−1^), monopotassium phosphate (1 g L^−1^), dipotassium phosphate (2 g L^−1^), glucose (8 g L^−1^), and magnesium sulphate (1 g L^−1^). The prepared substrate was subsequently sterilized in distilled water at 121 °C (15 psi) for 45 min.

Separately, a liquid nutrient medium was prepared by dissolving 3.0 g of glucose, 3.0 g of sucrose (Merck, Bratislava, Slovakia), 1.0 g of soy peptone, 2.5 g of yeast extract (Biolife, Monza, Italy), 1.0 g of monopotassium phosphate, 0.5 g of Epsom salt, 0.5 g of calcium chloride dihydrate, 0.01 g of ferrous sulphate heptahydrate, 1.0 mg of copper sulphate pentahydrate (Merck, Slovakia), and 0.1 g of thiamine hydrochloride (Sigma-Aldrich Co., St. Louis, MO, USA) in 1 L of distilled water. Three plugs (4 × 4 mm) of OS culture, excised from a Petri dish, were inoculated into this liquid medium. The flask was agitated on a rotary shaker at 120 rpm at 18 °C for 7 days to produce a homogenous liquid inoculum containing mycelial pellets.

Once the sterilized rice substrate had cooled to room temperature, 5 mL of the prepared liquid inoculum was aseptically added to the jar. The substrate was incubated at 18 °C for approximately 30 days to ensure full mycelial colonization throughout the rice matrix, alternating 12 h light and dark cycles. The samples were then dried at 40 °C in an APT Line dryer (Binder GmbH, Tuttlingen, Germany) and ground in an SM-100 mill (Retch Co., Haan, Germany).

The rice was prepared, sterilized, inoculated, and colonized by the fungus within the same specialized cultivation vessel—a borosilicate glass jar equipped with a special PTFE filter. This filter allows the rice to breathe while cooling prior to inoculation, while simultaneously protecting it from contamination from the ambient air.

### 2.2. Chemicals

The following chemicals, 2,2-diphenyl-1-picrylhydrazyl (DPPH), NaOH, HCl, glycine, CaCl_2_, NaHCO_3_, methanol 99.9% and trichloroacetic acid, were used as received from Sigma-Aldrich, Merck Millipore (St. Louis, MO, USA), and Mikrochem (Prešov, Slovak Republic) without further purification. Methanol 99.9% was used as received to measure the UV/Vis spectra. The number of chemical compounds in the extracts and fractions was monitored using thin-layer chromatography (TLC) on aluminum plates (Merck Millipore) with SiO_2_ 60 F254. Iodine vapour was used to visualize TLC stains. Trypsin (1:250, Thermo Fischer Scientific, Dreieich, Germany) was used to determine enzymatic activity. The azocasein substrate was prepared by the synthesis of casein with a diazonium salt, followed by recrystallization and drying with acetone before use.

### 2.3. Instruments

Two methods were employed to extract the mushroom samples: heat-reflux and ultrasonication (BANDELIN, Sonorex Digitec, P 140/560 W, f 35 KHz, DT 103H, Berlin, Germany). All extracts were evaporated to dryness using a rotary vacuum evaporator (IKA^®^ HB 10 digital, Staufen im Breisgau, Germany). The ^1^H and ^13^C NMR spectra (ppm) were measured on a 600 MHz spectrometer Varian VNMRS (Palo Alto, CA, USA). The IR spectra were measured on a Thermo Scientific spectrometer, NICOLET 6700 FT (Zevenhuizen, The Netherlands). The analyzed extracts were measured without further treatment. The UV/Vis spectra were measured at room temperature on a UV-1280 Multipurpose UV–Visible Spectrophotometer (Shimadzu, Kyoto, Japan) using 1 cm cuvettes containing 99.9% methanol (to measure antioxidant activity) and glycine-NaOH buffer (to measure the proteolytic activity). A pH metre (InoLab, pH 720, Weilheim, Germany) was used to determine the pH of the 0.1 M glycine/NaOH solution. To determine the enzymatic activity, samples were incubated in a BINDER Multifunctionaler Umluft-Wärmeschrank (230 V, 50/60 Hz, 2.70 kW, Tuttlingen, Germany).

### 2.4. Extract Preparations

Two extraction methods were applied to obtain biologically active constituents from OS strains. The methods employed were heat-reflux and ultrasound extraction (UE). Methanol was used as a solvent. First, 10.0 g of each sample 1OS–6OS was macerated in 50 mL of 99.0% methanol for 6 h in the dark. When the 1OS–6OS samples were extracted by heat-reflux, an additional 100 mL of 99.0% methanol was added to each sample. The sample mixtures were refluxed for 4 h and filtered, and the filtrates were evaporated to dryness using a rotary vacuum evaporator. UE of samples 1OS–6OS was performed by sonicating 10.0 g of each sample and 50 mL of 99.0% methanol in an ultrasonic bath for 1 h at 50 °C. Then, the samples were filtered, and the filtrates were evaporated to dryness using a rotary vacuum evaporator. The extraction yields of the individual samples obtained by the two extraction methods are shown in Table 2. The yields given in Table 2 are the average of the results obtained from three extractions.

### 2.5. Determination of Antioxidant Activity

In this study, antioxidant activity was determined by applying the DPPH radical scavenging method to alcoholic extracts of OS strains. The DPPH assay method is based on the reduction of DPPH, a stable free radical with a maximum absorption at 517 nm (which appears as a purple colour), to the DPPH-H form by reaction with antioxidants, resulting in decolorization (to a yellow colour) [25]. Antioxidant activity was measured using a freshly prepared 0.2 mM stock solution of stable DPPH radicals. The absorbance of the DPPH solution (prepared by mixing 1 mL of 0.2 mM stock with 1 mL of 99.9% methanol) was then measured at 517 nm against the corresponding blank solution (99.9% methanol).

To improve clarity, the measurement results are referred to as IC_50_ (the substrate concentration required to inhibit 50% of DPPH radical activity). All measurements of the scavenging of stable DPPH radicals were performed using extract concentrations of 10.0, 5.0, 2.5, 1.25, 0.625, 0.312 and 0.156 mg/mL. Table 3 summarizes the measured antioxidant activities of the 1OS–6OS methanol extracts.

The percent inhibition of the DPPH free radical was calculated based on a control reading using the following equation:

I (%) = (Abs_control_ − Abs_sample_)/Abs_control_) × 100,

where I (%) represents the inhibition of DPPH activity, Abs_control_ is the absorbance of the control reaction, and Abs_sample_ is the absorbance of the extract sample after 30 min of incubation in the dark at room temperature [26].

### 2.6. Determination of Proteolytic Activity

Nearly 70 years ago, Charney and Tomarelli [27] proposed the use of an azoprotein for the determination of proteolytic activity. This method relies on the reaction between a substrate and an enzyme at an optimum temperature/pH for a prescribed period. The intensity of the solution colour (red-orange at 440 nm) is a function of the quantity of the azoprotein digested, because all the residual proteins precipitate upon addition of trichloroacetic acid. Azocasein was chosen as the standard for these studies. A 0.1 M glycine/NaOH buffer with pH 8.3 was prepared. The pH of the prepared solution was determined by a pH metre. An enzyme stock solution of trypsin was prepared by weighing 100 mg of the enzyme into a 100 mL volumetric flask, and the enzyme was dissolved in a 0.001 M HCl solution containing 50 mM CaCl_2_. The solution in the flask was supplemented with a 0.001 M HCl solution. The resulting solution was stored in a refrigerator at 8 °C. The proteolytic activity of samples 1OS–6OS was determined using a substrate of the 1% azocasein solution in the 0.1 M glycine/NaOH buffer with pH 8.3 as follows: 2 mL of the 0.1 M glycine/NaOH buffer was added to 200 mg of individual fungi extracts, and the resulting mixtures were incubated in a dry oven at 37 °C for 1 h. Then, 2 mL of the 1% azocasein solution was added to each sample, and the resulting mixtures were incubated for 1 h at 37 °C. To ensure complete enzymatic activity, 3 mL of 10% trichloroacetic acid was added to each sample. The resulting precipitate was allowed to form for 20 min, and the solution was filtered; the absorbance of the prepared samples 1OS–6OS at 440 nm was measured against a blank. The blank sample was prepared similarly to the samples containing fungi, but the order of adding 10% trichloroacetic acid and 1% azocasein was changed. Table 4 summarizes the measured enzymatic activity compared to the trypsin activity expressed in U_trypsin_ units.

### 2.7. Biosynthesis of Silver Nanoparticles

**Figure 1:**
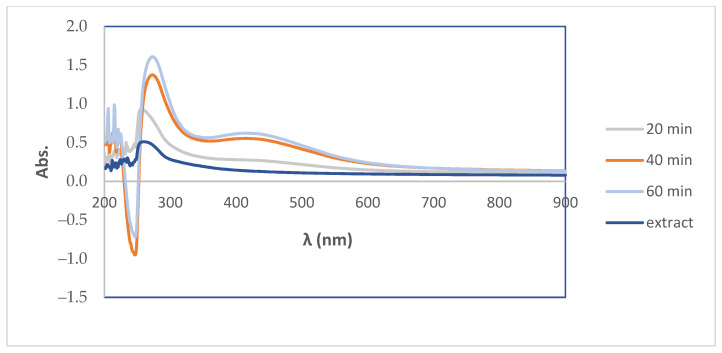
UV/Vis spectra showing the formation of silver nanoparticles using the extract of sample 5OS with silver ions (AgNO_3_). An increase in absorbance at 415 nm indicates nanoparticle formation over time. Spectra were recorded for the 5OS extract without AgNO_3_ (blue line) and after reaction with AgNO_3_ for 20 min (grey line), 40 min (orange line), and 100 min (light blue line).

The process of silver nanoparticle biosynthesis using extracts with silver ions was monitored spectrophotometrically in the range of 200–900 nm [16,28]. Samples for measurements were prepared from 10.0 mg of crude extract to which 2 mL of distilled water and 422 μL of 10.0 mM AgNO_3_ solution were added. The mixture was heated (80 °C) in a water bath and samples for measurements were taken at time intervals of 0, 20, 40, and 100 min. The samples were diluted with distilled water in a cuvette before the actual measurement. Figure 1 shows the results of spectrophotometric monitoring of the formation of silver nanoparticles from extracts prepared from sample 5OS by the heat-reflux method.

### 2.8. Antimicrobial Activity of Prepared Silver Nanoparticles

The antimicrobial effects of the extracts of samples 1OS–6OS were tested by the agar plate diffusion method [28,29]. Sample preparation was carried out by weighing 5 mg of all extracts into test tubes and dissolving the weights in 1 mL of distilled water. In this way, six test tubes with pure extract samples and another six test tubes with extract samples to which 422 μL of AgNO_3_ solution was added were prepared. All samples were prepared in triplicate. The tested bacteria—*Staphylococcus aureus* (SA) and *Escherichia coli* (EC)—were obtained from the Czech Collection of Microorganisms, Brno. The bacteria were cultured in BHI broth (Brain Heart Infusion broth, Oxoid) at 37 °C for 20 h.

After cultivation, the bacteria were diluted in phosphate-buffered saline to a concentration of 0.5–1.0 McFarland turbidity scale. This scale indicates the number of colony-forming units (CFUs) per mL. At a concentration of 0.5 to 1.0, approximately 1.5–3 × 10^8^ bacteria are present in 1 mL of suspension. Then, 1 mL of this suspension was inoculated into 100 mL of tempered liquid agar (Standard plate count agar, Oxoid). Subsequently, 20 mL of this agar was poured into 9 cm diameter Petri dishes. After solidification, wells with a diameter of 0.5 cm were cut into the agar and 50 μL of samples 1OS–6OS was applied to them. An aqueous solution of 30 μg/mL gentamicin sulfate was used as a positive control and distilled water as a negative control. The plates were incubated at 37 °C for 24 h. After incubation, the diameters of the formed inhibition zones were measured for each sample. The measured diameters were used to calculate the relative inhibition zone in percent, i.e., the value of % RIZD, for which we used the following relationship:

% RIZD = (IZD_SL_ − IZD_NEG_)/IZD_POZ_) × 100,

where IZD_SL_ represents the measured inhibition zone for a given sample, IZD_NEG_ denotes the inhibition zone of the negative control and IZD_POZ_ is the measured inhibition zone of the positive control. The calculated % RIZD value for individual samples served to express their antimicrobial activity [30].

### 2.9. NMR Spectroscopy

NMR spectra were recorded on a Varian VNMRS spectrometer (Palo Alto, CA, USA) operating at 599.87 MHz for ^1^H and 150.84 MHz for ^13^C. All measurements were carried out at 299.15 K unless otherwise stated. Pulse sequences from the Varian library were used. The ^1^H and ^13^C NMR spectra (ppm) of sample 2OS (15 mg) were measured in deuterated dimethyl sulfoxide (DMSO-d_6_, 0.6 mL, Merck Millipore). Proton and carbon assignments were performed for the ^1^H, ^13^C, ^1^H,^1^H-COSY, ^1^H,^13^C-HSQC, ^1^H, ^13^C-HMBC, and DEPT spectra. NMR spectra were processed and analyzed using MestreNova version 11.0.4–18998, 2017 (Mestrelab Research, Santiago de Compostela, Spain). The ratio of the major chemical compounds was determined based on the integrated intensity of the separated proton signals. The following proton signals were integrated: oleic acid (2.15 ppm, triplet, 2H); linoleic acid (2.73 ppm, triplet, 2H); and D-mannitol (3.61 ppm, doublet of a doublet, 2H).

### 2.10. Statistical Analysis

Statistical analysis was performed using GraphPad Prism version 10.6.1 (GraphPad Software, Boston, MA, USA). Data are presented as mean ± standard deviation (SD). Since the assumptions of parametric tests were not met and the sample size was small, statistical differences among groups were assessed using the Kruskal–Wallis test followed by Dunn’s multiple comparisons test. Differences were considered statistically significant at *p* < 0.05.

## 3. Results

### 3.1. Preparation of Extracts

Six specimens of OS cultured on two types of rice substrate, *Oryza sativa* var. *indica* and *Oryza sativa* var. *japonica*, were analyzed (Table 1). The prepared extracts, 1OS–6OS, were yellowish oils with a characteristic mushroom odour after evaporation of the solvent. For stability and further use, they were dissolved in ethanol and stored in a freezer at −20 °C.

Table 2 presents the extraction yields (%) of individual strains (1OS–6OS) obtained by reflux and ultrasound extraction methods. The highest extraction yield was obtained for sample 4OS using reflux extraction (9.86 ± 0.00%), whereas the lowest yield was observed for sample 2OS using reflux extraction (2.77 ± 0.008%). In general, reflux extraction provided higher yields compared to ultrasound-assisted extraction.

### 3.2. Measurement of Antioxidant Activity by the DPPH Method

Table 3 presents the antioxidant activity of samples 1OS–6OS in the concentration range of 10.00–0.156 mg/mL, compared with the IC_50_ value of gallic acid (75.6 μg/mL) (Table 4) [31]. Antioxidant activity was expressed as IC_50_ values (mg/mL) for extracts obtained by different extraction methods. Lower IC_50_ values indicate higher antioxidant activity. Among the reflux extracts, sample 5OS exhibited the strongest antioxidant activity (IC_50_ = 3.03 ± 0.01 mg/mL), whereas sample 6OS showed the weakest activity (IC_50_ = 6.06 ± 0.01 mg/mL). In general, reflux extracts demonstrated stronger antioxidant activity compared to ultrasound-assisted extracts. The values in Table 3 represent the mean antioxidant activity (%) obtained from three repeated absorbance measurements of the DPPH radical at 517 nm. IC_50_ values were calculated from the measured data using the linear function I (%) = f (concentration) in Microsoft Excel.

Table 4 shows the IC_50_ values for extracts prepared by reflux and UE. Results are presented as mean ± standard deviation (SD).

As shown in Table 4, the differences in antioxidant activity expressed as IC_50_ values vary considerably, which are influenced by the extraction method used and by the substrate used.

### 3.3. Measurement of Enzymatic Activity

The results of the evaluation of the proteolytic activity of six extracts prepared from OS strains are presented in Table 5. Table 5 summarizes the measured enzymatic activity compared to the trypsin activity expressed in U_trypsin_ units.

The highest proteolytic activity (expressed in U_trypsin_ units relative to the trypsin activity) was found for sample 3OS, cultured on the *Oryza sativa* var. *indica* substrate (101.75 U_trypsin_). Conversely, the lowest enzymatic activity was observed in sample 4OS, which was cultured on an *Oryza sativa* var. *japonica* substrate. As these results indicate, the choice of substrate during cultivation may influence the enzymatic activity of OS.

### 3.4. Formation of Ag Nanoparticles

The use of UV/Vis spectrophotometry showed that after adding AgNO_3_ to the extracts, an increase in absorbance was observed in the 415 nm region (Figure 1). The region from 400 to 500 nm represents the wavelength range with maximum absorption, which is typical for the formation of silver nanoparticles.

### 3.5. Antimicrobial Activity of Silver Nanoparticles

The antimicrobial activity of silver nanoparticle extracts (1OS–6OS) against *Escherichia coli* is summarized in Table 6. The highest relative inhibition zone diameter (% RIZD) was observed for sample 1OS (129.32 ± 0.58%), while the lowest activity was recorded for sample 6OS (86.17 ± 0.29%). In general, samples 1OS-4OS exhibited higher antimicrobial activity compared to samples 5OS and 6OS. Gentamicin sulfate (100%) was used as a positive control, while distilled water (0%) served as a negative control. % RIZD values are obtained as the average of three replicates.

### 3.6. NMR Spectrum Analysis

**Figure 2:**
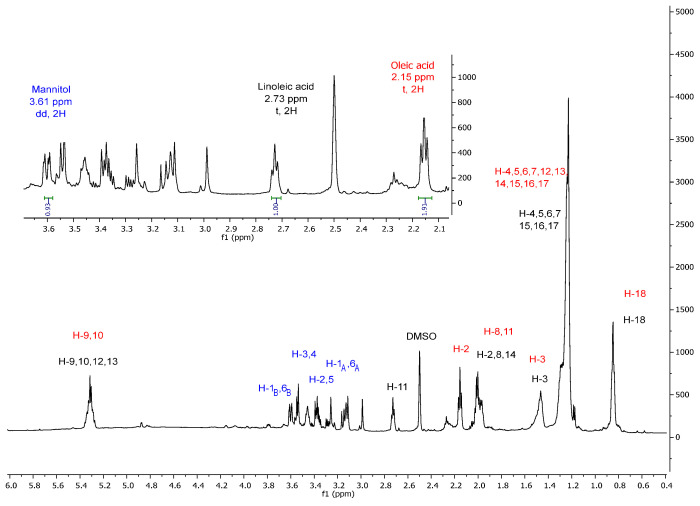
The ^1^H NMR spectrum of the methanol extract of sample 2OS measured in DMSO. Assignment of protons to individual chemical compounds based on integrated signal intensities for *Z*-oleic acid, linoleic acid and D-mannitol.

Figure 2 shows the proton spectrum of the 2OS extract. In the spectrum, signals are assigned to individual hydrogen atoms based on a thorough analysis of 2D NMR spectra. In the ^1^H NMR spectrum, the signals of the majority of substances whose chemical structure we managed to determine are distinguished by colour.

Detailed NMR analysis demonstrated that *Z*-oleic acid was the major compound in all extracts, followed by linoleic acid, while D-mannitol was present in the smallest amount and was not detected in samples 3OS-6OS (Table 7).

## 4. Discussion

Species of the genus *Cordyceps*, including *Ophiocordyceps sinensis*, *Cordyceps militaris*, *Cordyceps pruinosa* and *Cordyceps ophioglossoides*, are valuable traditional medicinal mushrooms that have been used for medicinal purposes for several centuries, especially in China, Japan and other Asian countries. Species of the genus *Cordyceps* are parasitic fungi associated with insect hosts. *Cordyceps* mushrooms are found at an elevation of 3600–4200 m above sea level, mainly in the Nepalese Himalayas, Tibet, Bhutan, Sikkim, Yunnan and other provinces of China [32]. In China, mushrooms of the genus *Cordyceps* have been used as a medicinal preparation of traditional Chinese medicine for centuries. So far, a considerable number of studies have been conducted that focused on analyzing the effects of *Cordyceps*, which include their antioxidant, antibacterial, immunomodulatory, antidiabetic, antitumor and many other effects [1].

### 4.1. Preparation of Extracts and Yield

Six specimens of OS cultured on two types of rice substrate, *Oryza sativa* var. *indica* and *Oryza sativa* var. *japonica*, were analyzed. The highest extraction yield was achieved for the 4OS sample grown on *Oryza sativa* var. *japonica* and extracted by heat-reflux, and the lowest extraction yield was obtained for the 2OS sample cultivated on *Oryza sativa* var. *indica* extracted using the same method.

The comparison of extraction methods in terms of yield shows that the use of heat-reflux is more effective than UE. We assume that the higher temperature during reflux may improve the solubility of the isolated chemical compounds from the extracted material, leading to a higher yield. The works of other authors, such as [33], also provide detailed insights into the suitability of various extraction methods for obtaining bioactive compounds from natural materials, including mushrooms. Conventional methods, including maceration, percolation, reflux, and ultrasonic extraction, have been used to extract bioactive compounds from plant materials for decades. Reflux is more effective than ultrasonic extraction or maceration, likely due to the higher temperature, which ensures greater solubility of substances in the given solvent; however, a disadvantage of this method is its use in the extraction of thermolabile substances [34]. For reflux extraction, significant differences in extraction yield were observed among the studied groups (Kruskal–Wallis test, *p* = 0.0050). A post hoc test with correction for multiple comparisons revealed a statistically significant difference between samples 2OS and 4OS (*p* = 0.0081), whereas no significant differences were detected among the remaining samples (*p* > 0.05). For the ultrasound-assisted extraction (UE), significant differences in extraction yield were also observed among the studied groups (Kruskal–Wallis test, *p* = 0.0051). A post hoc test with correction for multiple comparisons identified a statistically significant difference between samples 2OS and 3OS (adjusted *p* = 0.0083). No statistically significant differences were found in the remaining pairwise comparisons (all adjusted *p* > 0.05).

### 4.2. DPPH Scavenging Activity of OS Extracts

The extract 5OS prepared via heat-reflux and cultivated on the *Oryza sativa* var. *japonica* substrate exhibited the highest antioxidant activity, whereas sample 6OS, cultivated on *Oryza sativa* var. *indica*, exhibited the lowest antioxidant activity. Interestingly, among the individual strains, the rice substrate had the most striking effect on samples 5OS and 6OS, where the 5OS specimen cultivated on *Oryza sativa* var. *japonica* exhibited almost double the antioxidant activity of the 6OS specimen cultivated on *Oryza sativa* var. *indica*. Similar results were obtained for ultrasound-assisted extracts; the most striking differences were observed for the 5OS and 6OS sample strains. Sample 5OS exhibited 4.3-fold higher DPPH radical scavenging activity than the 6OS sample. Significant differences in IC_50_ values were observed among the reflux extracts (Kruskal–Wallis test, *p* = 0.0052), as well as among the ultrasound-assisted extracts (Kruskal–Wallis test, *p* = 0.0052). Among the reflux extracts, 5OS exhibited the lowest IC_50_ value (3.03 mg/mL), whereas 6OS showed the highest IC_50_ value (6.06 mg/mL). Similarly, for the ultrasound-assisted extracts, the lowest IC_50_ value was observed for 5OS (5.15 mg/mL) and the highest for 6OS (22.11 mg/mL), indicating the highest and lowest antioxidant activities, respectively. Post hoc analysis revealed statistically significant differences between these samples (Table 3).

The effect of the substrate was also most significant for the strains extracted by ultrasonication (samples 1OS and 2OS), where the sample grown on Japanese rice substrate had 1.8 times the DPPH radical scavenging activity of sample 2OS. Antioxidant activity and IC_50_ values of methanol extracts prepared by heat-reflux and UE of samples 1OS–6OS, determined by the DPPH method after 30 min incubation in the dark at 25 °C, are presented. Starch constitutes approximately 90% of rice grain and is therefore expected to affect rice quality. Starch is composed of linear amylose, a polysaccharide with low solubility in water, and branched amylopectin. Indica rice typically has a higher amylose content than japonica rice. *Oryza sativa* var. *japonica*, with low amylose content, appears to be more effective for the cultivation of 5OS and 6OS than the high-amylose *Oryza sativa* var. *indica*. The effect of different extraction methods on the IC_50_ showed that heat-reflux was the most efficient extraction method, producing IC_50_ values ranging from 3.03 to 6.06 mg/mL. Ultrasound-assisted extraction produced the highest IC_50_ of 6.10 to 22.11 mg/mL. These results show that heat-reflux for 4 h was the most efficient extraction method for leaching most of the substances with antioxidant activity into methanol [31]. The higher antioxidant activity of extracts prepared by heat-reflux compared to those prepared by UE is mainly due to differences in the chemical composition of the substances isolated using each extraction technique. The antioxidant activity of methanol extracts of OS is attributed primarily to phenolic compounds, polysaccharides, and nucleosides, particularly cordycepin. Phenolic compounds exhibit the highest capacity for direct free radical scavenging, while polysaccharides significantly contribute to overall antioxidant activity through the modulation of endogenous antioxidant systems [35,36,37]. Given the lower temperature (50 °C) used in UE, we can assume that fewer substances with antioxidant effects were isolated in extracts. The Japanese rice substrate had the most significant influence on samples 5OS and 6OS. Sample 5OS exhibited the lowest IC_50_, corresponding to the highest antioxidant potential. The same trend was observed for both extraction methods, i.e., the antioxidant activity decreased in the following order, (5OS) > (3OS, 4OS) > (1OS, 2OS), except for sample 6OS.

### 4.3. Enzymatic Activity of OS Extracts

Proteolytic enzymes (also termed peptidases, proteases and proteinases) are capable of hydrolyzing peptide bonds in proteins. They can be found in all living organisms, from viruses to animals and humans. Proteolytic enzymes have great medical and pharmaceutical importance due to their key role in biological processes and in the life-cycle of many pathogens [38]. Proteolytic enzyme treatments were first used in Germany in the 1960s for inflammation, osteoarthritis, autoimmune diseases, and viral infections. The products usually contain a mixture of pancreatin, papain, bromelain, trypsin, and chymotrypsin. Proteolytic enzymes also play an important role in digestion. A deficiency of digestive enzymes can be caused by a congenital disorder of absorption or enzyme production. The most common acquired cause is a disorder of the organ that produces the enzymes. Since the function of proteases is conditioned by specific conditions, a change in the pH of the stomach is often a problem. If the stomach acid is too acidic or, conversely, too alkaline, the enzymes are not activated and do not break down the proteins in food. This happens in ulcer disease or stomach cancer. Based on the above facts, we can conclude that the medical benefit of supporting proteolytic activity by using Cordyceps extracts is justified. *Cordyceps*, especially *Cordyceps militaris*, possesses proteolytic activity, meaning it contains enzymes that break down proteins. Proteolytic activity is crucial for various processes, including the tenderization of meat and potentially other biological, industrial and medicinal applications. Compared with our results, and in works by other authors, it was found that the proteolytic activity found in *Cordyceps* may play an important role in the therapy of various diseases [39,40,41].

Table 5 shows that the highest activity (expressed in U_trypsin_ units relative to the trypsin activity) was found for sample 3OS, cultured on the *Oryza sativa* var. *indica* substrate (101.75 U_trypsin_). Slightly lower activity (94.94 U_trypsin_) was found for sample 6OS, which was also cultured on the *Oryza sativa* var. *indica* substrate. The difference between the enzymatic activities of samples 6OS and 5OS was only 2.3 U_trypsin_. Sample 4OS, cultivated on *Oryza sativa* var. *japonica,* was less active. Minimal enzymatic activity was detected for samples 1OS (1.06 U_trypsin_) and 2OS (0.66 U_trypsin_) that were cultured on different substrates. Sample 1OS cultivated on *Oryza sativa* var. *japonica* had a slightly higher activity of approximately 0.4 U_trypsin_. Notably, distinct differences in the enzymatic activities of these samples were found. Certain *Oryza sativa* species are known to produce extracellular serine proteases [42,43,44,45,46], which are necessary for the breakdown of the chitin shell of insects and the degradation of chitin-binding proteins. These results suggest that substrate composition may influence the biological activity of individual fungal strains. The proteolytic activity of samples 1OS and 2OS was almost the same as that for samples 5OS and 6OS; thus, the substrate did not play as important a role as for the antioxidant activity. A different trend was observed for samples 3OS and 4OS, where the activity of an aqueous extract of sample 3OS grown on *Oryza sativa* var. *indica* was nearly twice that of sample 4OS cultivated on *Oryza sativa* var. *japonica*. We observed an increasing trend in the enzymatic activity of the strains: (1OS, 2OS) < (5OS, 6OS) < (3OS) (except for sample 4OS). This increased production of proteolytic enzymes may be useful for agricultural [46] and pharmaceutical [47] industries because appropriate quantities of enzymes can positively affect immunity, as well as suppress inflammation and some disease manifestations.

### 4.4. Preparation of Silver Nanoparticles (Ag NPs)

The use of UV/Vis spectrophotometry revealed that after adding AgNO_3_ to the extracts, an increase in absorbance was observed in the 415 nm region (Figure 1), which indicates the formation of silver nanoparticles (Ag NPs). The region from 400 to 500 nm represents the wavelength range with maximum absorption of silver nanoparticles [28]. By extending the reaction time between the extract and the AgNO_3_ solution, the absorbance in the 415 nm region increased. According to the Lambert–Beer law, an increasing concentration causes increasing absorbance, and this represents an increase in the ability of the extracts to form silver nanoparticles. The most significant increases in absorbance at 415 nm were seen after 100 min of heating, with the highest absorbances.

### 4.5. Antibacterial Activity of OS Extracts with Ag NPs

The prepared samples of 1OS-6O extracts were tested for their antimicrobial activity using the diffusion disk method on strains of SA and EC bacteria. After the incubation of the plates with the applied samples of 1OS–6OS and after their visual comparison, we can conclude that none of the 1OS–6OS extracts without the addition of AgNO_3_ solution showed an antibacterial effect on the strains of SA and EC bacteria. We recorded a change; there positive activity only in the case of samples of 1OS–6OS extracts after the addition of AgNO_3_ solution, i.e., the formation of nanoparticles, and on the strain of EC bacteria.

The % RIZD values ranged from 86.17 ± 0.29% (6OS) to 129.32 ± 0.58% (1OS) (Table 6). The highest activity was observed in sample 1OS, followed by samples 3OS, 2OS, and 4OS. Samples 5OS and 6OS exhibited lower % RIZD values compared to the other groups. The low standard deviation values indicate good reproducibility of the measurements (*n* = 3).

Because the data did not meet the assumptions for parametric testing and the sample size was small, differences among groups were evaluated using the Kruskal–Wallis test. Subsequent post hoc analysis using Dunn’s test with multiple-comparison adjustment identified a statistically significant difference only between samples 1OS and 6OS (adjusted *p* < 0.01), while no other pairwise comparisons reached statistical significance (*p* > 0.05).

In a study by Wang (2016) [15], the antimicrobial activity of biosynthesized silver nanoparticles against eight pathogens was determined by the diffusion method. The nanoparticles showed very good antimicrobial activity, but the cell filtrate from the mycelium of *Cordyceps militaris* did not form any inhibition zones. However, the combination of biosynthesized silver nanoparticles with *Cordyceps militaris* significantly inhibited the growth of bacteria. The results of this study are consistent with our findings. The study by Yang (2022) also describes the significant antibacterial effect of Ag NPs on a strain of *EC* compared to a strain of *SA* [48,49]. At the same time, it was found that the antibacterial effects of silver nanoparticles against Gram-negative bacteria were better than against Gram-positive bacteria. The difference between them lies in the thickness of the peptidoglycan layer of the cell wall, which suggests that the thicker cell wall of Gram-positive bacteria was the reason for the lower effectiveness of silver nanoparticles against them [16]. The conclusions of the study are again consistent with our results, as the silver nanoparticle extracts demonstrated antimicrobial activity against the *EC* strain, which is classified as a Gram-negative bacterium, but were ineffective against *SA*, which is a Gram-positive bacteria. Biogenic Ag NPs have gained considerable interest from researchers worldwide due to their antibacterial properties against a wide range of microorganisms, making them attractive for use in food contact materials (packaging materials). In addition, studies have indicated that biosynthesised Ag NPs are non-toxic to humans and highly effective against bacteria and fungi even at very low concentrations. The application of Ag NPs as antimicrobials in everyday products, including cosmetics, water purification, food processing, packaging technologies, pharmaceuticals and other nutritional supplements, has surpassed all other nanomaterials in the global market. Recent studies have reported the successful biosynthesis of Ag NPs of different sizes and shapes using various natural extracts [50,51,52,53]. Compared to other medicinal fungi (e.g., *Ganoderma lucidum*), the formation of Ag NPs is much easier, faster and less time-consuming when using extracts isolated from Cordyceps. As a result, the preparation of such Ag NPs is economically less demanding and more environmentally friendly.

From the perspective of the mechanism of the antibacterial effects of Ag NPs, we can assume based on the studied issues that: Ag+ released from Ag NPs induces ROS formation; Ag NPs interact with membrane proteins and change their functions; Ag NPs accumulate on the surface of the cell membrane and change their permeability; Ag NPs penetrate the cell, where they generate ROS, release Ag+ and damage DNA; the formed ROS can also damage DNA, cell membrane integrity and membrane proteins; and released Ag+ damages DNA and membrane proteins. The size and shape of nanoparticles also affect the antibacterial effects of Ag NPs. Triangular nanoplates have a significantly stronger biocidal effect (EC) than spherical or rod-shaped silver nanoparticles. When comparing the antibacterial activity of colloidal solutions of Ag NPs with diameters of 25, 35, 44 and 50 nm, it was found that the smaller the nanoparticles, the higher the antibacterial activity, and the reason is the easier penetration of smaller nanoparticles through the cell membrane [54].

### 4.6. NMR Analysis of OS Extracts

Based on a detailed analysis of infrared spectra, ^1^D and ^2^D NMR spectra, Z-oleic acid, linoleic acid, and D-mannitol were identified as the major chemical compounds in the methanol extracts of the samples 1OS–6OS (Figure 2). These chemical compounds were also identified in the works of other authors [55,56,57,58,59,60]. Proton and carbon assignments of major chemical compounds were based on analysis of ^1^H NMR, ^13^C NMR, correlation spectroscopy (COSY), heteronuclear single-quantum correlation spectroscopy (HSQC), heteronuclear multiple-bond correlation (HMBC), and distortionless enhancement by polarization transfer spectra (DEPT). The ^1^H NMR spectrum of the methanol extract of sample 2OS was measured in DMSO. The assignment of protons to individual chemical compounds was based on integrated signal intensities for *Z*-oleic acid, linoleic acid and D-mannitol (Figure 2, Table 7). Harvanová et al. [61] provided a detailed description of the NMR spectra (including chemical shifts, multiplicity of signals, and interaction constants) of the identified compounds. Depending on the extraction method (heat-reflux vs. UE), the ratios of major components present in the extracts changed only slightly.

In terms of the results achieved so far, future plans include a more detailed investigation of the formation of silver nanoparticles using OS extracts cultivated on different substrates. From the perspective of green synthesis of nanoparticles, future plans also include a detailed characterization of Ag NPs (their size and shape) and, of course, their stabilization in relation to the substrate used. Last but not least, the research is planned to be expanded to include testing of antimicrobial effects to include multiple strains of bacteria, and based on this, important conclusions can be drawn about the influence and importance of the substrate used in the cultivation of OS.

## 5. Conclusions

OS contains many biologically important chemical components with interesting pharmacological activity. The objective of the present study was to determine the antioxidant and enzymatic proteolytic activities of metabolites isolated from the fungal species OS, cultivated on two types of rice (*Oryza sativa* var. *indica* and *Oryza sativa* var. *japonica*). Samples were prepared using heat-reflux and ultrasonic extractions.

The comparison of extraction methods in terms of yield shows that the use of heat-reflux is more effective than UE. We assume that the higher temperature during reflux causes a better solubility of the isolated chemical compounds from the extracted material, leading to a higher yield.

The extract 5OS (IC_50_ 3.03 ± 0.01 mg/mL) prepared via heat-reflux and cultivated on the *Oryza sativa* var. *japonica* substrate exhibited the highest antioxidant activity, whereas sample 6OS, cultivated on *Oryza sativa* var. *indica*, exhibited the lowest antioxidant activity. Notably, among the individual strains, the rice substrate had the most striking effect on samples 5OS and 6OS, where the 5OS specimen cultivated on *Oryza sativa* var. *japonica* exhibited almost double the antioxidant activity of the 6OS specimen cultivated on *Oryza sativa* var. *indica*. Similar results were obtained for ultrasound-assisted extracts; the most striking differences were observed for the 5OS and 6OS sample strains. Sample 5OS exhibited 4.3-fold higher DPPH radical scavenging activity than sample 6OS.

The effect of the substrate was also most significant for the strains extracted by ultrasonication (samples 1OS and 2OS), where the sample grown on Japanese rice substrate had 1.8 times the DPPH radical scavenging activity of sample 2OS. Starch constitutes approximately 90% of rice grain and is therefore expected to affect rice quality. Starch is composed of linear amylose, a polysaccharide with low solubility in water, and branched amylopectin. Indica rice typically has a higher amylose content than japonica rice. *Oryza sativa* var. *japonica*, with low amylose content, appears to be more effective for the cultivation of 5OS and 6OS than the high-amylose *Oryza sativa* var. *indica*.

The highest activity (expressed in U_trypsin_ units relative to the trypsin activity) was found for sample 3OS, cultured on the *Oryza sativa* var. *indica* substrate (101.75 U_trypsin_). Slightly lower activity (94.94 U_trypsin_) was found for sample 6OS, which was also cultured on the *Oryza sativa* var. *indica* substrate. The difference between the enzymatic activities of samples 6OS and 5OS was only 2.3 U_trypsin_. Sample 4OS, cultivated on *Oryza sativa* var. *japonica*, was less active. Minimal enzymatic activity was detected for samples 1OS (1.06 U_trypsin_) and 2OS (0.66 U_trypsin_) that were cultured on different substrates. Sample 1OS cultivated on *Oryza sativa* var. *japonica* had a slightly higher activity of approximately 0.4 U_trypsin_.

The highest proteolytic activity was found for OS grown on *Oryza sativa* var. *indica* (3OS 101.75 U_trypsin_).

Based on spectrophotometric measurements, the extract of sample 5OS cultivated on *Oryza sativa* var. *japonica* (prepared by heat-reflux) demonstrated the ability to biosynthesize silver nanoparticles, which were identified by an increase in absorbance at 415 nm. The use of UV/Vis spectrophotometry revealed that after adding AgNO_3_ to the extracts, an increase in absorbance was observed in the 415 nm region, which indicates the formation of Ag NPs.

Antimicrobial activity was shown only by the extracts of samples 1OS–6OS after the formation of silver nanoparticles; pure extracts of samples 1OS–6OS did not show any effectiveness against the tested pathogenic bacteria (EC) and (SA). The best antimicrobial activity against the EC strain was shown by sample 1OS, whose average value of % RIZD was 129.32 ± 0.58%; antimicrobial activity was found to be proven only against the strain of Gram-negative bacteria. This issue of biological testing of nanoparticles remains open, as it is necessary to optimize the testing conditions from the point of view of the stability of nanoparticles.

The chemical structure of the compounds from the alcohol extracts was determined by ^1^D and ^2^D NMR and IR spectroscopy. Unsaturated fatty acids, Z-oleic acid and linoleic acid, as well as D-mannitol, were identified as the major components of the extracts.

## Figures and Tables

**Table 1:** Labelling *Ophiocordyceps sinensis* strains according to the substrate.

Production Strain Catalog Number	*Oryza sativa* var. *japonica*	*Oryza sativa* var. *indica*
MFTCCB026/0216	1OS	2OS
MFTCCB025/0216	4OS	3OS
MFTCCB023/0216	5OS	6OS

Strain labels (1OS–6OS) correspond to Ophiocordyceps sinensis production strains cultivated on different rice substrates. *Oryza sativa* var. *japonica and Oryza sativa* var. *indica* were used as cultivation media.

**Table 2:** Comparison of extraction yields obtained using different extraction methods.

Method of Extraction	Yield (%)					
	1OS	2OS	3OS	4OS	5OS	6OS
Reflux	6.38 ± 0.006	2.77 ± 0.008	8.10 ± 0.005	9.86 ± 0.000	7.00 ± 0.005	8.61 ± 0.000
UE	3.68 ± 0.015	2.89 ± 0.005	6.82 ± 0.015	3.79 ± 0.000	4.75 ± 0.005	5.16 ± 0.006

The data were analyzed using the statistical software GraphPad Prism version 10.6.1 (GraphPad Software, Boston, MA, USA). Results are presented as mean ± standard deviation. Because the data did not meet the assumptions for parametric analysis and the number of observations in each group was small (*n* = 3), group comparisons were performed using the Kruskal–Wallis test.

**Table 3:** Antioxidant activity of samples 1OS–6OS determined by the DPPH assay.

Sample Concentration (mg/mL)	Antioxidant Activity (%)											
	Reflux						UE					
	1OS	2OS	3OS	4OS	5OS	6OS	1OS	2OS	3OS	4OS	5OS	6OS
10.00	87.4	76.8	87.9	82.0	89.0	73.3	58.0	29.2	72.5	75.7	78.3	23.3
5.000	59.4	47.9	67.1	79.2	88.3	49.9	37.4	22.8	39.0	41.9	62.2	8.8
2.500	27.7	40.5	41.9	60.7	71.9	38.7	8.0	13.2	22.2	38.7	58.6	0
1.250	19.8	12.5	22.3	26.0	52.7	9.2	1.7	4.9	14.5	18.8	9.2	0
0.625	0.7	5.5	23.8	7.9	33.6	4.3	0	0.9	6.4	0	8.1	0
0.312	1.0	4.5	13.0	6.2	14.8	0	0	0	2.7	0	1.1	0
0.156	0	4.3	11.7	4.0	12.4	0	0	0	4.1	0	0	0

Values are expressed as mean ± SD (*n* = 3).

**Table 4:** IC_50_ values of reflux and UE determined by the DPPH assay.

Sample	IC_50_ (mg/mL)	
	R	UE
1OS	5.22 ± 0.006 ^ab^	8.32 ± 0.015 ^ab^
2OS	5.82 ± 0.006 ^ab^	15.30 ± 0.006 ^ab^
3OS	4.45 ± 0.006 ^ab^	6.67 ± 0.006 ^ab^
4OS	4.36 ± 0.006 ^ab^	6.10 ± 0.006 ^ab^
5OS	3.03 ± 0.006 ^a^	5.15 ± 0.006 ^a^
6OS	6.06 ± 0.006 ^b^	22.11 ± 0.006 ^b^

IC_50_ gallic acid 75.6 (±0.3) μg/mL. Different letters within the same extraction method indicate statistically significant differences according to Dunn’s multiple comparisons test following the Kruskal–Wallis test (*p* < 0.05). Lower IC_50_ values indicate higher antioxidant activity.

**Table 5:** Enzymatic activity of samples 1OS–6OS determined using a 1% azocasein substrate at 37 °C.

Sample	A (440 nm) ^a^	V_trypsin_ (μL) ^b^	m_trypsin_ (μg) ^c^	U_trypsin_^d^
1OS	0.064	169.00	42.25	1.06
2OS	0.045	105.66	26.41	0.66
3OS	0.502	1629.00	407.25	101.75
4OS	0.300	955.66	238.91	59.73
5OS	0.458	1482.33	370.58	92.64
6OS	0.469	1519.00	379.75	94.94

^a^ Values represent the mean absorbance obtained from three independent measurements. ^b^ Determined using Microsoft Excel by extrapolation of the linear function V (μL) = f (m). ^c^ Calculated using Microsoft Excel by extrapolation of a linear function based on silver nanoparticles. ^d^ Calculated based on the specific activity of trypsin (250 U/mg).

**Table 6:** Relative inhibition zone values (% RIZD) of tested silver nanoparticle extracts 1OS–6OS.

Sample (% RIZD) ^a^					
1OS	2OS	3OS	4OS	5OS	6OS
129.32 ± 0.58 ^a^	125.00 ± 1.00 ^ab^	127.68 ± 0.58 ^ab^	120.00 ± 1.00 ^ab^	91.00 ± 0.00 ^ab^	86.17 ± 0.29 ^b^
Gentamicin sulphate 100					
Distilled water 0					

Values are presented as mean ± standard deviation (SD), *n* = 3. Different superscript letters indicate statistically significant differences between groups according to Dunn’s post hoc test following the Kruskal–Wallis test (*p* < 0.05).

**Table 7:** Ratio of the major chemical compounds of the mixture in 1OS–6OS sample extracts prepared by heat-reflux and ultrasound extraction determined from the ^1^H NMR spectra by integration of the distinct signals for *Z*-oleic acid, linoleic acid and D-mannitol.

Sample	Reflux	UE
	Ratio of *Z*-Oleic Acid:Linoleic Acid:D-Manitol	
1OS	4.80:2.41:2.79	4.82:2.48:2.70
2OS	5.10:2.65:2.25	4.81:2.82:2.37
3OS	6.06:3.94:0	6.38:3.62:0
4OS	5.88:4.12:0	5.85:4.15:0
5OS	4.50:5.50:0	4.84:5.15:0
6OS	6.70:3.30:0	6.10:3.90:0

## Data Availability

All data generated or analyzed during this study are included in this published article.
